# Synthesis, Characterization, and *In Vitro* Evaluation of New Ibuprofen Polymeric Prodrugs Based on 2-Hydroxypropyl Methacrylate

**DOI:** 10.3797/scipharm.1204-14

**Published:** 2012-12-10

**Authors:** Mirzaagha Babazadeh, Maryam Sheidaei, Sara Abbaspour, Ladan Edjlali

**Affiliations:** Department of Chemistry, Faculty of Science, Tabriz Branch, Islamic Azad University, Tabriz, Iran.

**Keywords:** Ibuprofen, 2-Hydroxypropyl methacrylate, Polymeric prodrugs, Controlled release systems, Polymerization

## Abstract

The present research work describes the synthesis and evaluation of new acrylic-type polymeric systems having degradable ester bonds linked to ibuprofen as materials for drug delivery. Ibuprofen was linked to 2-hydroxy-propyl methacrylate by an activated ester methodology in a one-pot procedure with a high yield. The resulting material was copolymerized with either 2-hydroxyethyl methacrylate or methyl methacrylate (in 1:3 mole ratios) by the free radical polymerization method, utilizing azoisobutyronitrile at 65–70 °C. The characterization of the resulting products by FTIR, ^1^H NMR, ^13^C NMR, DSC, and elemental analysis confirmed their synthesis successfully. Ibuprofen release from the obtained polymers was preliminarily evaluated at different buffered solutions (pH 1, 7.4, and 10) into dialysis bags to show the capacity of prodrugs to release the drug under hydrolytic conditions. Detection of hydrolysis by UV spectroscopy at selected intervals showed that the drug can be released by selective hydrolysis of the ester bond at the side of the drug moiety. The release profiles indicated that the hydrolytic behavior of polymers is strongly based on the polymer hydrophilicity and the pH value of the hydrolysis solution. The results suggest that these polymers could be useful in controlled release systems.

## Introduction

Ibuprofen, 2-(4-isobutylphenyl)propanoic acid, is a member of the non-steroidal anti-inflammatory drugs (NSAIDs) with well-known anti-inflammatory, antipyretic, and analgesic properties. It is most commonly administered orally such as tablets, gel, pellets, and syrup dosage forms and is rapidly absorbed to reach its maximal plasma concentration. However, it has a short biological half-life, which means that frequent doses are required to maintain the therapeutic efficacy over extended time periods [1, 2].

The therapeutic use of NSAIDs is often restricted by the necessity to deliver the drug to the specific sites of the target organ or tissue. Also, the use of NSAIDs is limited by their irritant side effects on the gastro-intestinal mucosa because of the presence of their free carboxylic group and by their frequent poor water solubility [3]. One particular approach towards an improved use of NSAIDs for therapeutic applications is the preparation of polymeric prodrug backbones which can contain drugs in a physically bound (dissolved, dispersed, included, or adsorbed) state or by true chemical linkages along the polymer backbone or as side groups. These latter systems, in which drugs are delivered by chemically or biologically induced cleavage of the covalent bonds, increase water solubility, stability, and therapeutic efficiency of drugs while reducing the required doses, side effects, and toxicity of drugs by controlling the rate, duration, and site of release [4–6].

Acrylic-type polymers are an important class of the macromolecules used in drug delivery systems. The advantages of acrylic-based macromolecular prodrugs have been reviewed by Dumitriu in 1989 [7]. These systems do not form toxic by-products during their biodegradation, which have tendency to swell, when they come in contact with biological environment. In recent years, ibuprofen and some of the NSAIDs such as indomethacin, naproxen, ketoprofen, mefenamic acid, diclofenac, and fenoprofen have been attached to various polymer backbones and their *in vitro* evaluation has been studied widely [8–22].

Literature studies show that many reports about the preparation and *in vitro* evaluation of 2-hydroxyethyl methacrylate (HEMA) polymeric prodrugs containing NSAIDs have been published, but the application of 2-hydroxylpropyl methacrylate (HPMA) polymers in controlled release systems of NSAIDs has been investigated in only a few articles.

The main aim of this work is the synthesis, characterization, and *in vitro* controlled release study of new polymeric carriers based HPMA-containing ibuprofen pendants. First, 2-{[2-(4-isobutylphenyl)propanoyl]oxy}propyl methacrylate (IbuPMA), as a new acrylic-type polymerizable derivative of ibuprofen was synthesized by the esterification methodology between ibuprofen and HPMA in a one-pot procedure. The novelty of this synthesis is that, in just one step, the hydrolyzable-ibuprofen pendant group acrylic monomer is obtained with a high yield. The obtained IbuPMA was then copolymerized with either 2-hydroxyethyl methacrylate (HEMA) or methyl methacrylate (MMA) by the free radical polymerization technique, and their detailed molecular structures were characterized via FTIR, NMR, DSC, and elemental analysis. The release of ibuprofen from the obtained polymeric prodrugs was carried out *in vitro* by hydrolysis in buffered solutions into cellophane dialysis bags at various pH values, and the quantity of the released drug was detected by UV spectroscopy at selected intervals. The effects of neighbouring groups and pH values on the release of ibuprofen from the synthesized novel polymeric prodrugs are discussed.

## Results and Discussion

### Synthetic route for preparation of IbuPMA

**Fig. 1:**
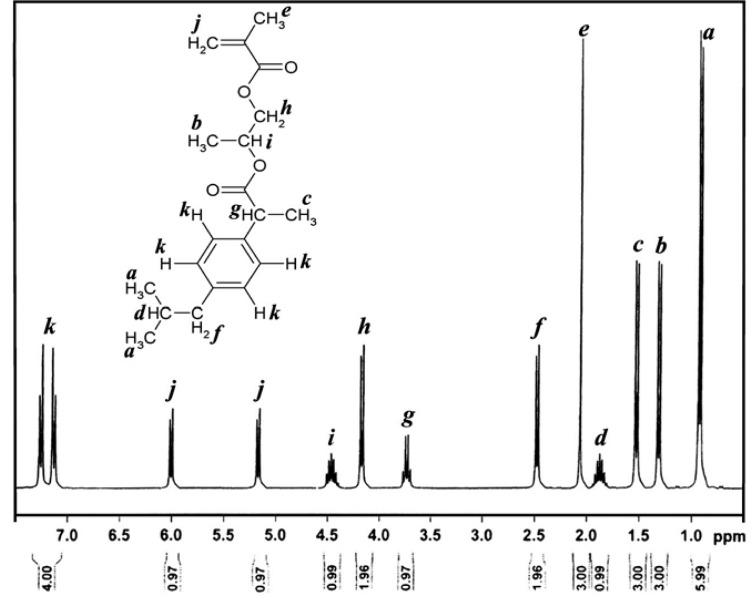
^1^H NMR spectrum of IbuPMA in CDCl_3_.

**Fig. 2:**
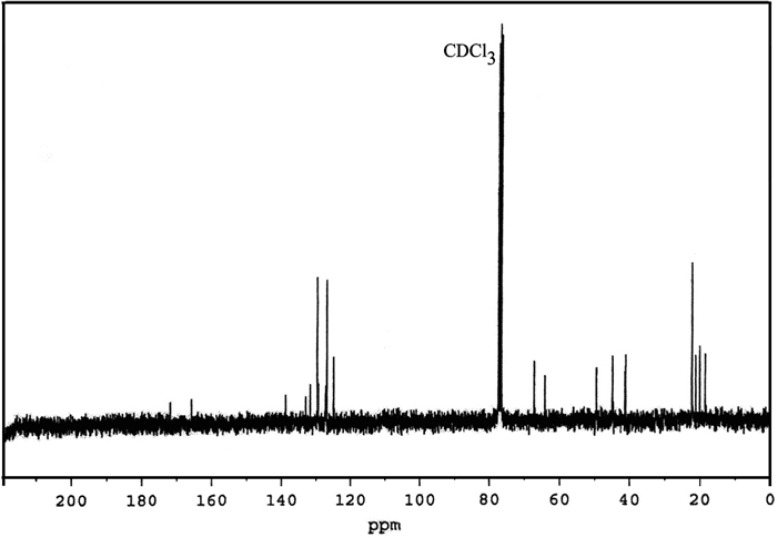
^13^C NMR spectrum of IbuPMA in CDCl_3_.

IbuPMA was easily synthesized by direct esterification of ibuprofen with HPMA in the presence of *N, N*-dicyclohexylcarbodiimide (DCC) in CH_2_Cl_2_ solution (Scheme 1). The hydroxyl group of HPMA reacted with the carboxyl group of ibuprofen and the resulting water was absorbed by DCC to produce *N, N*-dicyclohexylurea (DCU) as a white precipitate. The white precipitate was isolated and the solvent was evaporated to give IbuPMA as a stable monomer. The ^1^H and ^13^C NMR spectra of IbuPMA are shown in Figs. 1 and 2.

### Synthesis and characterization of polymeric prodrugs

The drug-containing monomer, IbuPMA, was easily copolymerized with HEMA and MMA in dried DMF solution by the free radical technique at 65–70 °C using AIBN as an initiator (Scheme 2). The resulting copolymers were colorless, amorphous, and soluble in DMSO and DMF, but insoluble in water and alcohols. The conversions of monomers to the related copolymers were determined gravimetrically after exhaustive drying of the isolated copolymer samples.

**Fig. 3:**
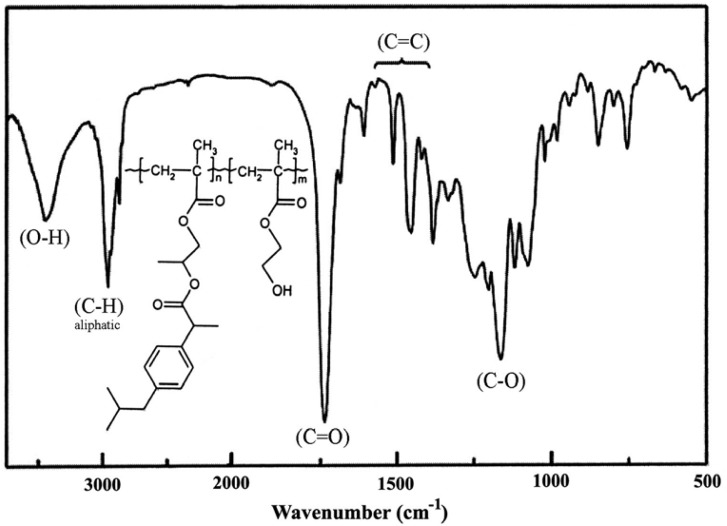
FT-IR spectrum of poly(IbuPMA-*co*-HEMA).

**Fig. 4:**
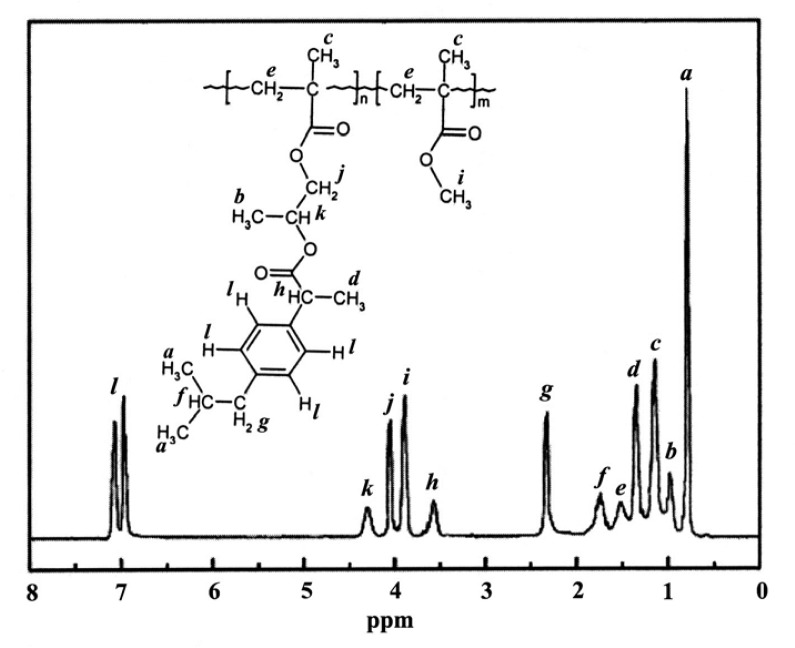
^1^H NMR spectrum of poly(IbuPMA-*co*-MMA) in DMSO-*d*_6_.

The formation of the desired polymeric prodrugs was confirmed by FT-IR and NMR spectroscopy. Typically, the FT-IR spectrum of poly(IbuPMA-*co*-HEMA) and the ^1^H NMR spectrum of poly(IbuPMA-*co*-MMA) with the corresponding signal assignment are shown in Figs. 3 and 4, respectively. In these Figs., the methacrylic double bond signals are missing while those of the aliphatic main chain appear.

The resulting spectral characteristics of functional groups of all the synthesized copolymers having ibuprofen substituents from FT-IR, ^1^H NMR, and ^13^C NMR spectroscopies are given in Table 1.

One parameter in the characterization of the polymeric prodrugs is the determination of the molecular weight distribution and the average molecular weights. In relation to the polymeric prodrugs, the rate of hydrolysis in the heterogeneous system can be controlled by the structure of the polymer substrates and their molecular weight. The rate of hydrolysis is lowered as the molecular weight increases. The weight and number-average molecular weights of the synthesized polymeric prodrugs were estimated by the gel permeation chromatography (GPC) instrument. The obtained values are shown in Table 2.

The mole compositions of the polymeric prodrugs were determined from ^1^H NMR spectroscopic data and elemental analysis of prodrugs (Table 3). In the past few decades, ^1^H NMR spectroscopy and elemental analysis have been established as powerful tools for the determination of copolymer compositions because of their simplicity, rapidity, and sensitivity [23]. In this work, the obtained mole compositions from ^1^H NMR data and elemental analyses were relatively in good agreement. Also, the drug content of the copolymers was calculated. The results showed that this method permits the incorporation of 18–21 wt. % of ibuprofen into copolymer structures. The drug content of the copolymers and the required doses of the prodrugs, which are equivalent to 300 mg of ibuprofen, are calculated in Table 3.

**Fig. 5:**
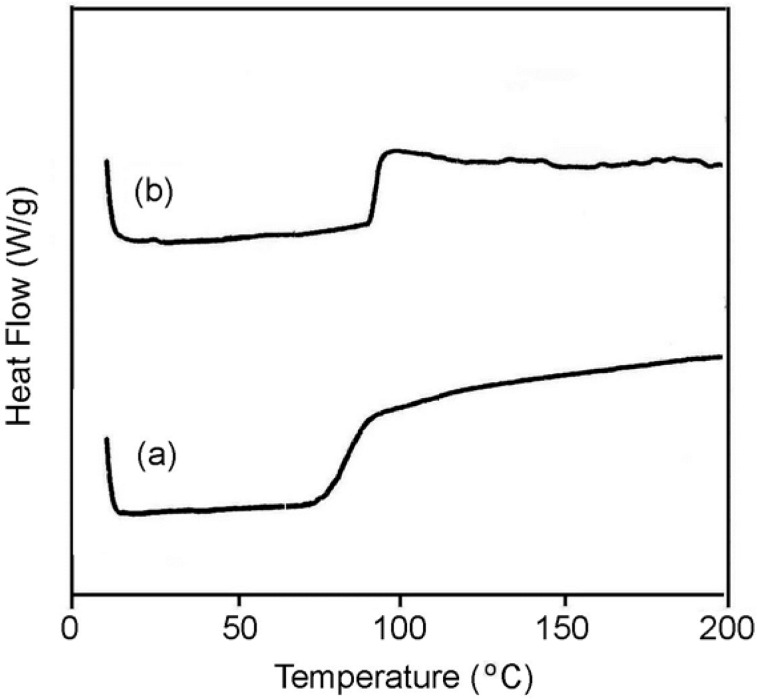
DSC curves of (a) poly(IbuPMA-*co*-HEMA) and (b) Poly(IbuPMA-*co*-MMA).

The thermal behavior of a polymer is important in relation to its properties for controlled release and its ability to be processed into suitable dosage forms [24, 25]. Differential scanning calorimetry (DSC) was used to determine the thermal properties of the polymeric prodrugs. The glass transition temperature (*T*_g_) value was determined from the DSC thermodiagrams (Fig. 5). The obtained *T*_g_ values are collected in Table 3. DSC analysis indicated that the polymeric prodrugs were amorphous and no crystallinity was observed within the evaluated temperature from 10 to 200 °C. Also, the synthesized polymeric prodrugs showed only a single *T*_g_, showing the absence of formation of a mixture of homopolymers or the formation of a block copolymer. Comparing the resulting *T*_g_ values of the polymeric prodrugs with that of methacrylic homopolymers, such as poly(propyl methacrylate) (*T*_g_ = 35 °C), showed that these values are quite high, which is attributed to the rigid ibuprofen pendant groups that decrease the mobility of the polymer chains.

### Drug release by hydrolysis of polymeric prodrugs

It has been widely demonstrated that the side chain hydrolysis of drug pendent polymers depends on the strength and chemical nature of the drug polymer chemical bonds, the structure of the polymer, and the surrounding conditions. The hydrolysis of a linkage is also dependent on its distance from the polymer backbone. The length and hydrophilicity of the spacer unit between the drug and polymer chain can affect the release rate. The *in vitro* hydrolysis behavior of polymeric prodrugs was studied in physiological conditions (aqueous phosphate or hydrochloric acid buffers, at 37 °C). As the polymers were not soluble in water, they were dispersed in buffer solution and the hydrolysis was performed in a heterogeneous system. The hydrolysis was carried out in cellophane membrane bags permeable to low molecular weight compounds. The released drug passed through the high molecular weight polymers into the external buffer solution and was determined by a UV spectrophotometer. Two hydrolysable ester bonds are present in polymers. Detection of the hydrolyzing solution by UV spectrophotometer showed that only the ester bond between the drug moiety and methylene group is hydrolyzed during the reaction time. The IR spectroscopic data and melting point measurements of the residue corresponded to the free drug. The direct ester linkage between the main chain of the polymer and methylene group does not undergo hydrolysis under mild conditions. This can be related to the steric hindrance of bulk polymer chains, which decreases the bond mobility [26, 27]. The hydrolysis mechanism of polymers prodrugs in different pH conditions is shown in Scheme 3.

**Fig. 6:**
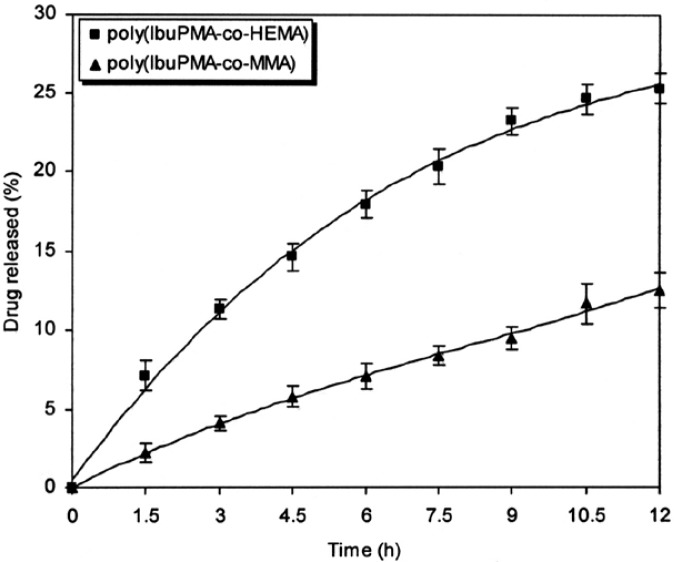
Percent of ibuprofen released from polymeric carriers as a function of time at hydrochloric acid buffer (pH 1) and 37 °C.

Fig. 6 shows the ibuprofen release from polymeric prodrugs as a function of time under mild conditions at pH 1 (HCl buffer). The drug-release rate from polymeric prodrugs at this pH is very low. It seems that at acidic media (pH 1), the carboxyl group of hydrolyzed ibuprofen will be protonated (pKa 4–5) and its aqueous solubility will be lower than in alkali media, where the acid group is deprotonated. Also, the hydrolysis of the ester in acidic media is actually an equilibrium reaction, as ester formation is also catalysed by acid. The position of this equilibrium is governed by a range of factors such as the solubility and neighbouring effect of side groups in polymeric prodrugs. As the system is heterogeneous with hydrophilic and hydrophobic domains, and the hydrolysis product (ibuprofen) is poorly soluble in the aqueous phase at this pH, it is most likely to be present in the hydrophobic domains. As the water content here is low, this may drive the local equilibrium towards ester formation inside these domains and the hydrolysis in this medium would be expected to happen only at the surface of the hydrophobic domains. Therefore, hydrolysis of the polymeric prodrug with hydrophilic domains in buffer solutions must be, rather than polymeric prodrug with hydrophobic domains.

**Fig. 7:**
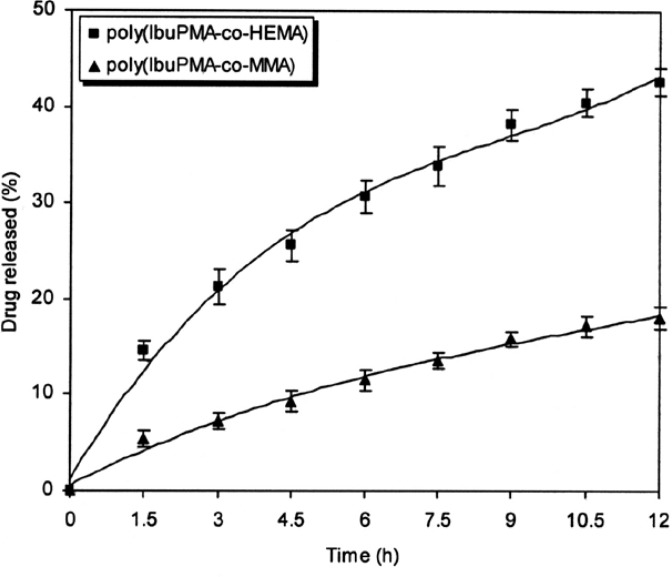
Percent of ibuprofen released from polymeric carriers as a function of time at phosphate buffer (pH 7.4) and 37 °C.

**Fig. 8:**
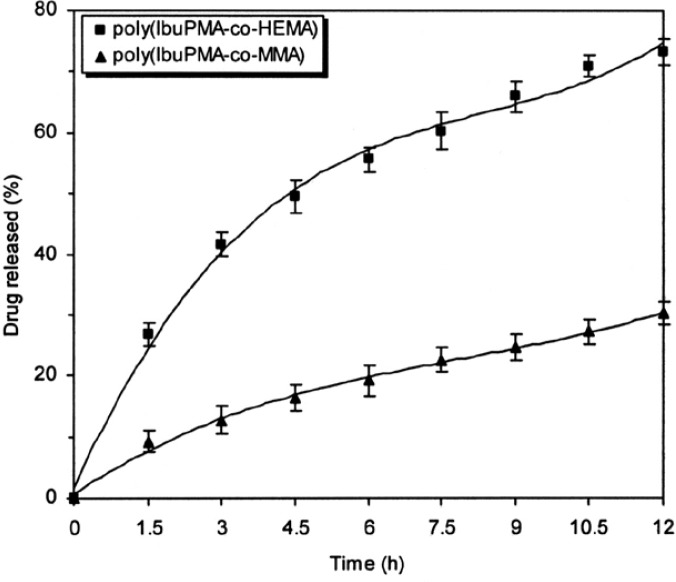
Percent of ibuprofen released from polymeric carriers as a function of time at phosphate buffer (pH 10) and 37 °C.

Figs. 7 and 8 show the ibuprofen release from polymeric prodrugs as a function of time under mild conditions at pH 7.4 and pH 10 (KH_2_PO_4_–Na_2_HPO_4_ buffer), respectively. A study of the drug-release profiles shows that the release rate of ibuprofen from polymeric prodrugs in alkaline medium is higher than the drug-release rate in acidic conditions. The results show that with passing polymeric prodrugs from acidic media to a slightly alkaline pH, the labile bonds are more accessible to hydrolysis and the polymers are easily degraded to release ibuprofen in alkaline pH values.

Finally, the neighbouring groups can affect the drug-release rate. As shown in Figs. 6–8, the hydrolysis rate of poly(IbuPMA-*co*-HEMA) is higher than poly(IbuPMA-*co*-MMA). It seems that poly(IbuPMA-*co*-HEMA) has hydrophilic HEMA units and therefore, is rapidly hydrolyzed from poly(IbuPMA-*co*-MMA) containing hydrophobic MMA units.

## Experimental

### Materials

Ibuprofen was purchased from Aldrich and was recrystallized from an acetone/hexane mixture (1/1 v/v). HPMA, HEMA, and MMA were obtained from Merck and purified by distillation under reduced pressure to remove inhibitors. *N, N*-Dicyclohexylcarbodiimide (DCC) and 4-(dimethylamino)pyridine (DMAP) were obtained from Merck and employed as received. Azoisobutyronitrile (AIBN) was obtained from Fluka and recrystallized twice from methanol. Dichloromethane (CH_2_Cl_2_) and was dried over calcium hydride prior to use. *N, N*-dimethylformamide (DMF) was dried over anhydrous magnesium sulphate for two days and later with phosphoric anhydride overnight. After drying, DMF was distilled under reduced pressure. Other solvents were employed without previous purification. All other chemicals were reagent grade or purer.

### Instrumental measurements

FT-IR spectra were recorded on a Shimadzu 4300 spectrophotometer. ^1^H and ^13^C NMR spectra were recorded on a Bruker 400 MHz spectrometer in CDCl_3_ or DMSO-*d*_6_ solution. The amount of released ibuprofen was determined by a 2100 Shimadzu UV spectrophotometer at the adsorption maximum of the free drug in aqueous buffered solutions (λ_max_=264 nm) using a 1-cm quartz cell. The values of number-average molecular weight (*M*_n_), weight-average molecular weight (*M*_w_), and the polydispersity index of polymers were determined with a Maxima 820 gel permeation chromatography (GPC) instrument consisting of two GPC columns (Ultrastyragel 10^4^ Å and 10^3^ Å) connected in series (Mobile phase: DMF, run time: 50 min, column temperature: 50 °C, detector: refractive index model 410). Well-characterized polyethylene oxide was used in the calibration within the range of *M*_w_ between 2.6×10^3^ and 8.8×10^5^ g mol^−1^. Elemental analyses were carried out with a Heareus CHN-ORAPID instrument. The glass transition temperatures (*T*_g_)s were determined with a Perkin-Elmer DSC7 thermal analyzer system at a heating rate of 10 °C min^−1^ under 20 mL min^−1^ of dry nitrogen gas over a temperature range from 10 °C to 200 °C. The *T*_g_ values were taken at the midpoints of the heat flow changes.

### Preparation of 2-{[2-(4-Isobutylphenyl)propanoyl]oxy}propyl methacrylate (IbuPMA)

In a two-necked flask, ibuprofen (4.12 g, 20 mmol) and DMAP (1.25 g, 10 mmol) were dissolved in 40 mL of dry CH_2_Cl_2_. The flask was cooled until 0 °C and a solution of DCC (4.12 g, 20 mmol) dissolved in 40 mL of dry CH_2_Cl_2_ was added dropwise into the flask solution at 0 °C. Then, HPMA (2.5 g, 17.36 mmol) was dissolved in 20 mL of dry CH_2_Cl_2_ and added dropwise to the flask solution at the mentioned temperature. The reaction mixture was vigorously stirred at 0 °C for 1 h and returned slowly to room temperature. The mixture was stirred at room temperature about 24 h and filtered for the removal of the white precipitation of *N,N*-dicyclohexylurea (DCU). The organic layer was sequentially extracted three times by 10 wt % of NaHCO_3_, twice by HCl (2 N), once by deionised water, and finally by a saturated NaCl solution. The extracted organic solution was dried over MgSO_4_ anhydrous and the solvent was removed under vacuum to obtain a yellow liquid. After it was set aside at room temperature overnight, a white precipitate appeared. It was removed and the final product was obtained as a yellowish liquid in a yield of 85.7%. FT-IR, cm^−1^: 3073 (C-H aromatic), 3040 (C-H vinylic), 2950, 2890 (C-H aliphatic), 1715, 1735 (C=O ester), 1637 (C=C vinylic), 1600, 1480 (C=C aromatic), 1200, 1170 (C-O). ^1^H NMR (400 MHz, CDCl_3_, TMS): δ 0.91 (d, 6H, -CH(CH_3_)_2_), 1.30 (d, 3H, -OCH(CH_3_)-), 1.51 (d, 3H, -Ar-CH(CH_3_)-), 1.88 (m, 1H, -CH_2_CHMe_2_), 2.07 (s, 3H, =CCH_3_), 2.48 (d, 2H, Ar-CH_2_-), 3.72 (q, 1H, Ar-CH(CH_3_)-), 4.17 (d, 2H, -OCH_2_-), 4.45 (m, 1H, -OCH(CH_3_)-), 5.17 (d, 1H, CH_2_=), 5.97 (d, 1H, CH_2_=), 7.1-7.3 (dd, 4H, aryl-H). ^13^C NMR (100 MHz, CDCl_3_, TMS): δ 18 (1C, -Ar-CH(CH_3_)-), 20 (1C, -CH(CH_3_)-O-), 21 (2C, -CH(CH_3_)_2_), 22 (1C, -CH(CH_3_)_2_), 40 (1C, =C(CH_3_)-), 45 (1C, Ar-CH_2_-), 49 (1C, -Ar-CH(CH_3_)-), 64 (1C, -CH(CH_3_)-O-), 67 (1C, -OCH_2_-), 125 (1C, CH_2_=C-), 132 (1C, -CH_2_=C(CH_3_)-), 126, 129, 131, 139 (6C, aromatic carbons), 166 (1C, CH_2_=C(CH_3_)-COO-), 172 (1C, -Ar-CH(CH_3_)-COO-). Anal. Calcd. for C_20_H_28_O_4_ (332 g mol^−1^): C, 72.29; H, 8.43. Found: C, 71.94; H, 8.23%. UV–Visible (dioxane, 25 °C): λ_max_ = 233.5 nm.

### Copolymerization of IbuPMA with acrylic monomers (general procedure)

In two Pyrex glass ampoules, a mixture of IbuPMA (3.32 g, 10 mmol) and AIBN (0.16 g, 1 mmol) was dissolved in 15 mL of dry DMF. Then, HEMA (3.90 g, 30 mmol) was added into the first ampoule and MMA (3.00 g, 30 mmol) was added into the second ampoule, respectively. The ampoules were then degassed, sealed off under vacuum, maintained at 65–70 °C in a water bath and shaken by a shaker machine for about 48 h. After this time, the viscous solutions were separately poured from the ampoules into a large amount of cooled methanol as non-solvent. The precipitate polymers were collected, washed with non-solvent several times, and dried under vacuum at room temperature. The yields of the obtained polymeric prodrugs are presented in Table 2.

### Method of hydrolysis

The polymer-drug conjugates were dried under vacuum at room temperature and sieved with a 200-mesh sieve. Each of the dried polymer-drug conjugates (20 mg) was poured into 5 ml of aqueous buffered solution (pH 1, 7.4 and 10) at 37 °C and the mixture was conducted into a cellophane membrane dialysis bag. The bag was closed and transferred into a flask containing 25 mL of the same buffer solution maintained at 37 °C. The external solution was continuously stirred and a 3 mL sample was removed at selected intervals and 3 mL of buffer was replaced. The quantity of released drug was analyzed by means of a UV spectrophotometer at 264 nm and determined from the calibration curve obtained previously under the same conditions. In each concentration measurement, an equal volume of fresh buffer was added to the hydrolysis solution and the dilution of the hydrolysis solution occurred during the hydrolysis process. Therefore, for the calculation of the mean concentration of the released drug, each concentration measurement was corrected according to the following equation:

$${\text{c}}_{\text{n}}={\text{c}}_{\text{n.meas}}\frac{\Delta \text{V}}{{\text{V}}_{\text{total}}}\sum_{\text{i}=1}^{\text{i}=\text{n}-1}{\text{c}}_{\text{i.meas}}$$

where, n indicates the n^th^ concentration measurement, V_total_ is the total volume of hydrolysis solution (25 ml), ΔV is the withdrawn volume at each measurement (3 mL), C_n.meas_ is the obtained drug concentration at the n^th^ measurement, and C_n_ is the corrected drug concentration in the hydrolysis solution due to the introduction of a volume ΔV of buffer.

Finally, three parallel tests were done for one sample in each pH media and then both the average value and error bar were calculated.

### Characterization of hydrolysis products

Twenty milligrams of the polymer-drug adduct was dispersed into 20 mL of buffered solution (pH 10) and maintained at 37 °C. After 24 h, the hydrolysis solution was sampled, neutralized with HCl (1 N), and the solvent was removed under vacuum. The resulting crude product was treated with 10 mL of acetone and heated. The suspension was then filtered and the acetone solution was evaporated under reduced pressure. The residue was characterized by melting point measurement, IR, and ^1^H NMR spectroscopy, and showed that the hydrolysis product is ibuprofen. mp 78 °C. IR (KBr), cm^−1^: 3480–2400 (O-H), 3100 (C-H aromatic), 2960 (C-H aliphatic), 1728 (C=O), 1600, 1450 (C=C aromatic). ^1^H NMR (400 MHz, CDCl_3_, TMS): δ 0.9 (d, 6H), 1.5 (d, 3H), 1.8 (m, 1H), 2.4 (d, 2H), 3.7 (q, 1H), 7.0-7.2 (dd, 4H), 11.5 (s, 1H).

## Conclusion

In this work, IbuPMA as an acrylic-type polymerizable derivative of ibuprofen was directly synthesized from the reaction between HPMA and ibuprofen by the esterification method. Then, the polymeric prodrugs containing ibuprofen pendent groups were synthesized by the free radical polymerization of IbuPMA with acrylic monomers such as HEMA and MMA. The structures of the synthesized IbuPMA and polymeric prodrugs were characterized by various spectroscopy techniques. A study of DSC thermodiagrams of the polymeric prodrugs indicated that the presence of ibuprofen pendant groups in polymer structures increases the polymer’s rigidity with increasing glass transition temperature. Hydrolysis of polymeric prodrugs was carried out in conditions similar to physiological conditions and the results showed that the introduction of hydrophilic units along the polymer chain improve the hydrolytic behavior. Also, the resultant release profiles of drug from prodrugs showed that the synthesized polymeric prodrugs were pH-sensitive polymers. Therefore, the studied polymers in the present investigation can be used in the prolongation of transit time and are useful as drug carriers for the development of pH-sensitive polymeric prodrugs. As the main purpose of polymeric prodrugs is the achievement of controlled drug release or slow release, application of these polymers as a drug delivery system is expected after *in vivo* examinations.

## Figures and Tables

**Sch. 1:**
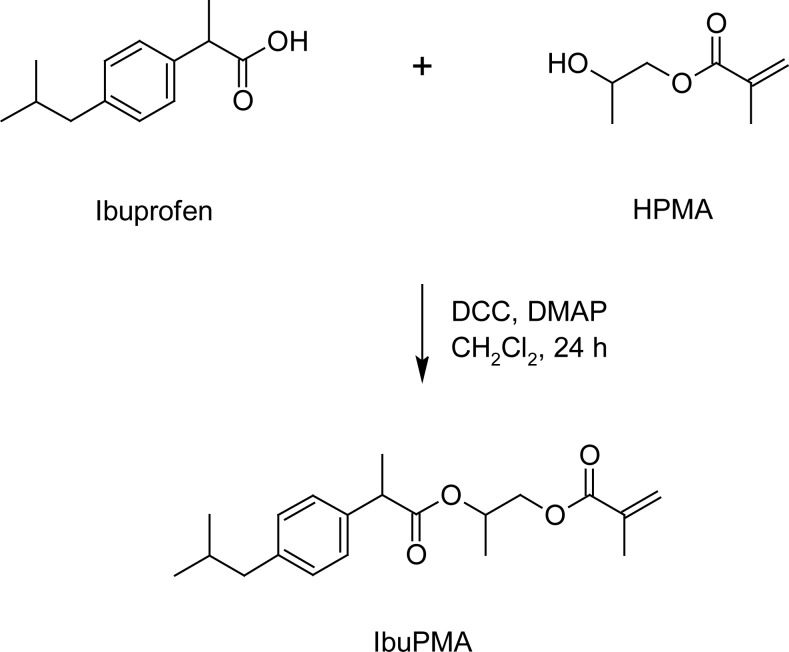
The synthesis route of acrylic-type derivatives of ibuprofen (IbuPMA).

**Sch. 2:**
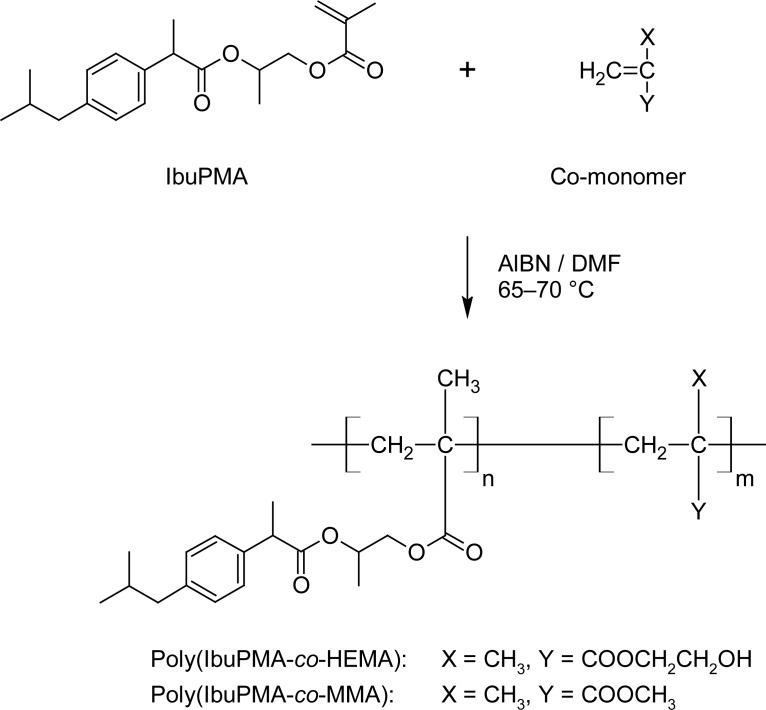
Copolymerization of IbuPMA with either HEMA or MMA.

**Sch. 3:**
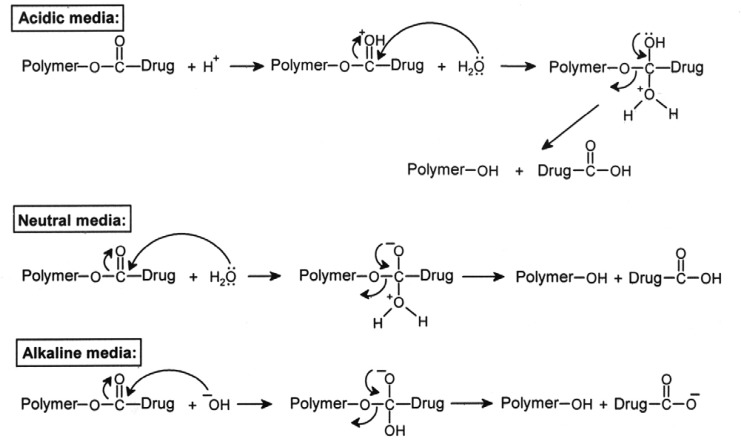
The hydrolysis mechanism of polymeric prodrugs in different pH medias.

**Tab. 1.** Spectral characterization of the synthesized polymeric prodrugs

**Sample**	**Functional group**	**^1^H NMR (ppm)**	**^13^C NMR (ppm)**	**FT-IR (cm^−1^)**
Poly(IbuPMA-*co*-HEMA)	OH	5.5	–	3400
Poly(IbuPMA-*co*-MMA)	OMe	3.8	60	1100
All polymers	COO	–	172	1735
	CH_2_O	4.2	63	1150
	CHO	4.4	65	1150
	Ph	7.0–8.0	126, 129, 135, 139	1520, 1480

**Tab. 2.** The yields, molecular weights, and glass transition temperature (*T*_g_) values of the polymeric prodrugs

**Sample**	**Yield (%)**	***M*_n_**	***M*_w_/*M*_n_**	***T*_g_ (°C)**
Poly(IbuPMA-*co*-HEMA)	67.6	31320	1.7	82
Poly(IbuPMA-*co*-MMA)	73.4	29563	1.9	94

**Tab. 3.** Elemental analysis, mole compositions, and the drug content of the polymeric prodrugs

**Sample**	**C (%)**	**H (%)**	**n (%)**	**m (%)**	**D (%)**	**P.D. (**g**)**
Poly(IbuPMA-*co*-HEMA)	64.18	8.07	30	70	18.6	1.6
Poly(IbuPMA-*co*-MMA)	67.63	8.27	33	67	20.5	1.5

n…content of drug containing monomer (IbuPMA); m…content of co-monomer (HEMA or MMA) in Scheme 2; D…content of ibuprofen in the polymeric prodrug; P.D.…the prodrug doses equivalent to 300 mg of ibuprofen.
